# The optimal childbearing age and birth spacing in china: a multicenter retrospective cohort study

**DOI:** 10.1186/s12889-025-24466-6

**Published:** 2025-08-30

**Authors:** Zhijia Zhao, Hui Zhu, Hongyi Liu, Yanming Chen, Qiong Zhu, Jie Cai, Jin Xu, Qinkang Lu, Lindan Ji

**Affiliations:** 1 Medical Record Statistics Room, Affiliated People’s Hospital of Ningbo University, Ningbo, China; 2 School of Public Health, Health Science Center, Ningbo University, Ningbo, China; 3 Department of Clinical Medicine, Health Science Center, Ningbo University, Ningbo, China; 4 Department of Pediatrics, Affiliated People’s Hospital of Ningbo University, Ningbo, China; 5 Department of Reproductive Medicine, Ningbo Women and Children’s Hospital, Ningbo, China; 6 Department of Ophthalmology, Affiliated People’s Hospital of Ningbo University, Ningbo, China; 7 Department of Biochemistry and Molecular Biology, School of Basic Medical Sciences, Health Science Center, Ningbo University, Ningbo, China; 8 Department of Science and Education, Affiliated People’s Hospital of Ningbo University, Ningbo, China

**Keywords:** Maternal age, Birth spacing, Adverse outcomes, Joinpoint regression analysis

## Abstract

**Background:**

We aimed to comprehensively analyze the impact of both maternal age and birth spacing on adverse maternal and neonatal outcomes.

**Methods:**

A total of 151,301 pregnancies in China from January 1, 2010, to December 31, 2021 were included, and 8,222 subjects matched their primary and second pregnancy information. Join point regression was used to analyze the temporal trends of adverse outcomes with maternal age and birth spacing.

**Results:**

The mean maternal age at delivery rose from 27.1 years to 28.5 between 2010 and 2021, and the average annual percentage change in advanced maternal age (≥ 35 years) was 1.2%. The optimal childbearing age (with the fewest adverse outcomes) appears to be 27 years. Compared with women aged 27 years, both those aged < 27 years and those aged > 27 years exhibited a significantly higher risk of anemia, low birth weight (LBW), preeclampsia, and preterm birth. In addition, we found that the optimal primiparous childbearing age was 26 years old and birth spacing was 3 years, < 3 years increased the risk of FGR, oligohydramnios, placenta previa, preeclampsia, LBW, preterm birth and premature rupture of membranes (PROM). While birth spacing > 3 years significantly increased the risks of anemia, gestational diabetes mellitus (GDM), placenta previa, preeclampsia, thyroid dysfunction (TD), preterm birth and PROM. The subgroup analysis revealed that the common adverse outcomes increased with longer birth spacing in pregnant women < 26 years, while these adverse outcomes showed lowest prevalence rates at 3-year birth spacing in pregnant women > 26 years.

**Conclusions:**

The age of childbearing continues to be delayed, and we are in favor of having children at relatively optimal ages and spacing.

**Supplementary Information:**

The online version contains supplementary material available at 10.1186/s12889-025-24466-6.

## Introduction

In recent years, the phenomenon of women giving birth at an advanced maternal age (AMA) of 35 years or older has become increasingly prevalent on a global scale [1]. Data from a cross-sectional study revealed that the proportion of advanced-age primiparas in the United States (US) rose from 6.6% during 1995–1999 to 10.0% during 2015–2019 [2]. Additionally, the percentages of advanced-age multiparous women reached 18.3% in the same period [3]. In Japan, the proportion of births to AMA women increased markedly from 9.8% in 1996 to 28.5% in 2016 [4]. In parallel, Japan’s mean age at first childbirth also saw an increase from 25.7 years in 1975 to 30.7 years in 2018 [5]. China has also witnessed a similar trend, with this phenomenon being significantly influenced by its fertility policies. During the “one-child policy” era (2011–2013), deliveries by women of advanced maternal age (AMA) accounted for 12.5% of all births. Following the implementation of the “universal two-child policy” (2017–2019), this proportion rose to 21.1% [6]. With the rollout of the “three-child policy,” the number of births in China is likely to increase significantly, as some families may choose to have a third child. This development could help improve the country’s demographic structure [7]. However, maternal age has been a well-known independent risk factor for pregnancy complications and adverse perinatal outcomes [8–10]. A large number of studies have shown that both adolescent pregnancy (under 20 years of age) and advanced maternal age (35 years or old) are associated with a significantly elevated risk of adverse pregnancy outcomes, including miscarriage, preterm birth, and low birth weight (LBW) [11–13]. In the context of China’s post-three-child policy era, childbearing age has emerged as a critical public health issue with direct policy implications for maternal-infant health and population sustainability.

Studies have shown that, in addition to maternal age, birth spacing is also a factor influencing adverse pregnancy outcomes, and it is affected by maternal age [14, 15]. Birth spacing, defined as the interval between two successive births, has received increasing scholarly attention given its substantial implications for fertility and maternal-child health outcomes. The World Health Organization (WHO) recommends a minimum inter-birth interval of 33 months between consecutive live births, or a minimum of 24 months between a birth and the subsequent pregnancy [16], in order to reduce the likelihood of adverse pregnancy outcomes in subsequent pregnancies [17–19]. A retrospective cohort study based on data from the Jiangsu Maternal Child Information System indicates that the risk curve for low birth weight (LBW) in relation to interpregnancy interval (IPI) exhibited an L-shaped pattern, with the lowest point occurring at approximately 40 months, corresponding to a predicted absolute risk of 0.02. Shorter interpregnancy intervals increased the predicted absolute risk, peaking at 0.035 for an IPI of 2 months. Longer interpregnancy intervals showed a stable absolute risk of around 0.025 [20]. Long birth spacing are also linked to adverse pregnancy outcomes. A systematic review and dose-response meta-analysis showed that birth spacing ≥ 60 months is associated with an increased risk of preeclampsia and gestational diabetes mellitus (GDM). Dose-response analyses further confirmed the presence of J-shaped relationships (P *non-linear* < 0.001–0.009) [21]. And a Cross-Sectional Study in Northwest China has shown that long IPI (≥ 120 months) was associated with a higher risk of LBW (RR = 1.54, 95% CI: 1.01–2.34) and PTB (RR = 1.73, 95% CI: 1.08–2.76) than an IPI of 18–23 months [22].

However, although current studies have shown that both age and birth spacing affect pregnancy outcomes, they have not analyzed the optimal age and spacing for childbearing. In this study, we aimed to comprehensively analyze the impact of both maternal age and birth spacing on adverse maternal and neonatal outcomes. We determined the optimal maternal age and the most appropriate birth spacing in the context of the changing fertility situation in China today.

## Materials and methods

### Patient selection and information

In this retrospective study, data from 151,301 pregnant women who delivered in multiple healthcare centers in Ningbo, Zhejiang Province, from January 1, 2010, to December 31, 2021, were systematically collected. The participating institutions comprised the Affiliated People’s Hospital of Ningbo University, Ningbo Women and Children’s Hospital, and Beilun People’s Hospital, which are three prominent tertiary hospitals representative of the healthcare system in Ningbo city. The three hospitals account for over 40% of all maternal and child deliveries in Ningbo City. Clinical information pertaining to maternal demographics, adverse complications and outcomes was obtained from the electronic medical record system. Inclusion criteria: [1] Age 20–49 years; [2] Singleton live birth. Exclusion criteria: [1] Cases with miscarriage occurring before 28 weeks of gestation; [2] Women with pre-pregnancy diagnosis of diabetes, hypertension, polycystic ovary syndrome (PCOS), or mental disorders; [3] Women with twin or assisted reproductive technology (ART) pregnancies; [4] Missing important data, such as number of pregnancies or deliveries. After screening, a total of 151,301 eligible subjects were finally included for subsequent analysis.

**Fig. 1:**
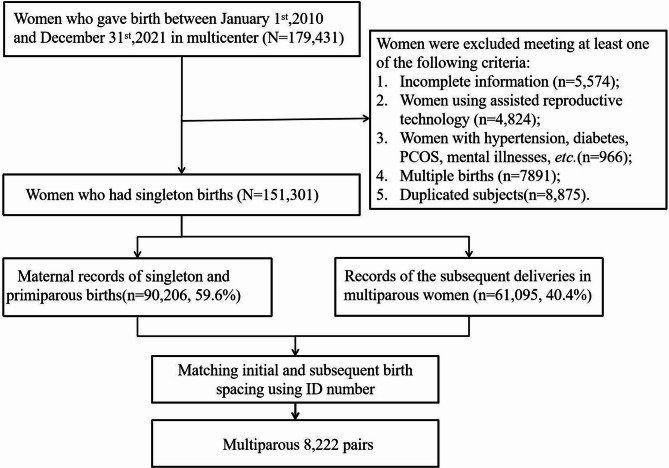
Flow chart of the study participants

As illustrated in the flowchart (Fig. 1), a total of 179,431 women gave birth at the aforementioned three hospitals between January 1, 2010, and December 31, 2021. Participants with incomplete data, such as age, parity or gravidity, were excluded from the analysis initially. Women with twin or multiple pregnancies [23], PCOS [24, 25], pre-existing diabetes [26, 27], hypertension [28], mental health disorders [29, 30], or those who conceived by ART [31] were excluded from the study, as these factors are well-documented to substantially increase the complexity of pregnancy health and are significantly correlated with a range of adverse pregnancy outcomes. Duplicate data were also removed. Ultimately, a total of 151,301 pregnancies were included in the initial phase of the study to analyze the association between maternal age and adverse outcomes. Similarly, we screened the data of multiparous women, removing duplicate and incomplete data and excluding patients with ART, PCOS, etc. Subsequently, we matched the information of primiparous and multiparous women based on their identity card information, using the identity card number as the unique identifier and employing the R 4.3.1 for data matching. If a patient had three or more deliveries in the above-mentioned hospitals, only the data from the first and second deliveries were retained. Based on this, we further calculated the birth spacing among the 8,222 subjects and analyzed the association between birth spacing and adverse outcomes.

### Data collection and outcome assessment

Since adolescent pregnancy was defined as 10–19 years of age and AMA as ≥ 35 years at delivery [32], we divided the subjects into five maternal age groups: <20 years old, 20–24 years old, 25–29 years old, 30–34 years old and ≥ 35 years old. The duration of birth spacing equaled the maternal age at second delivery minus the age at first delivery. Referring to the birth spacing recommended by the WHO [33], birth spacing was divided into six groups, < 2 years, 2 years, 3 years, 4 years, 5 years and > 5 years.

The following common adverse outcomes related to maternal and fetal health were used as outcome variations: anemia, GDM, fetal growth restriction (FGR), oligohydramnios, pregnancy-induced hypertension (PIH), placenta previa (poor placental implantation near or above the cervix), polyhydramnios, preeclampsia, thyroid dysfunction (TD), LBW (birth weight < 2500 g), macrosomia (birth weight ≥ 4000 g), placental abruption, postpartum hemorrhage (PPH), preterm birth (< 37 weeks of delivery) and PROM [34]. Here, anemia was defined as low hemoglobin (Hb) concentrations based on trimester-specific cutoffs (Hb < 11.0 g/dl during the first trimester, Hb < 10.5 g/dl during the second trimester, Hb < 11.0 g/dl during the third trimester) [35]. GDM was diagnosed by the one-step criterion suggested by the WHO, namely, fasting glucose level ≥ 5.1 mmol/L or 1 h 75 g oral glucose tolerance test (OGTT) ≥ 10.0 mmol/L or 2 h OGTT ≥ 8.5 mmol/L. FGR was defined as fetal growth below the 10th percentile for gestational age and not reaching the genetically predetermined growth potential [34]. Blood pressure greater than 140/90 mmHg after 20 weeks of gestation in the absence of proteinuria was diagnosed as PIH, whereas such a blood pressure in the presence of proteinuria was diagnosed as preeclampsia. PPH referred to blood loss ≥ 500 mL after vaginal delivery or ≥ 1000 mL after cesarean delivery. PROM was a report of watery leakage from the vagina, confirmed by sterile speculum examination and by the observation of either fluid accumulation in the posterior vaginal fornix or direct leakage from the cervical canal with pressure from uterine fundus or a cough attempt, was evidence of rupture. Placental abruption was diagnosed based on the presence of vaginal bleeding, with ultrasonography aiding in the differential diagnosis. All adverse results were diagnosed according to the criteria of the International Classification of Diseases, Tenth Revision (ICD-10): anemia (O99.008), GDM (O24.900), FGR (P05.900), oligohydramnios (O41.000 × 001), PIH (O13.x00 × 001), placenta previa (O44.000 × 003), polyhydramnios (O40.x00 × 001), preeclampsia (O14.900), TD (O99.217), LBW (P05.001), macrosomia (P08.100 × 001), placental abruption (O45.900), PPH (O72.101), preterm birth (P07.300 × 002) and PROM (O42.000).

### Statistical analysis

Continuous variables were described as mean ± standard deviation (SD), while categorical variables were described as n (%). The chi-square test was used to compare the maternal characteristics among different maternal age groups and different birth spacing groups. Join point regression was used to analyze the temporal trends of adverse outcomes with maternal age and birth spacing. Here, the temporal trends refer to changes with age and birth spacing. This analysis integrated multiple linear segments at “junctions” to evaluate whether the changes in time trends were statistically significant. In this model, proportions of adverse outcomes were used as the dependent variables, and maternal age/birth spacing was used as the independent variable.

Logistic regression analysis was used to verify the recommended age and birth spacing of join point regression analysis. The recommended age at delivery (27 years old) and the birth spacing between deliveries (3 years) were used as references to evaluate the effects of being younger or older than that age and birth spacing on adverse outcomes, after adjusting for parity, gravidity, and the histories of abnormal pregnancy. The odds ratio (OR) and 95% confidence interval (95%CI) were calculated. In this study, the occurrence of any adverse pregnancy outcome during the second delivery was counted to calculate the overall incidence of adverse outcomes. This overall incidence was used as the dependent variable to predict its probability using a nomogram. The predictors included maternal age, history of adverse outcomes, parity, and birth spacing. Nomograms are constructed based on multivariate regression models, with each indicator’s score determined by the magnitude of its regression coefficient. The regression coefficient reflects the extent of the indicator’s contribution to the outcome event. Therefore, by comparing the scoring ranges and weights of different indicators on the nomogram, we can roughly determine which indicators have a greater impact on the outcome. Data analyses were performed using SPSS software version 24.0 (Chicago, IL), R software (version 4.1.0) and the join point regression program 4.9.1.0 of the National Cancer Institute April 2022 (http://surveillance.cancer.gov/joinpoint). *P* values < 0.05 were considered statistically significant.

## Result

A total of 151,301 participants were included in this study. The pregnancy information (age, parity, delivery mode) and childbirth information (fetal gender, fetal height, and weight) of these participants are summarized in Table 1. The average maternal age of the participants was 28.5 years, with the highest proportion (42.4%) in the 25-29-year-old group.

**Table 1:** The characteristics of subjects among different maternal age groups

Variables	All	< 20 years old	20–24 years old	25–29 years old	30–34 years old	≥ 35 years old	*P*
Number (%)	151,301	4206 (2.8)	25,441 (16.8)	64,197 (42.4)	39,311 (26.0)	18,146 (12.0)	
Age (y)	28.5 ± 4.9	18.1 ± 1.1	22.5 ± 1.4	27.1 ± 1.4	31.7 ± 1.4	37.3 ± 2.3	< 0.001
Parity	2.0 ± 0.9	1.4 ± 0.6	1.7 ± 0.8	1.8 ± 0.9	2.3 ± 0.9	2.7 ± 0.8	< 0.001
Obstetric history							< 0.001
Primipara	90,206 (59.6)	3692 (87.8)	18,932 (74.4)	47,238 (73.6)	16,884 (42.9)	3460 (19.1)	
Multipara	61,095 (40.4)	514 (12.2)	6509 (25.6)	16,959 (26.4)	22,427 (57.1)	14,686 (80.9)	
P/M	1.48	7.18	2.91	2.79	0.75	0.24	
Delivery mode							< 0.001
Vaginal deliveries	83,914 (55.5)	3260 (77.5)	17,110 (67.6)	37,983 (59.2)	18,802 (47.8)	6659 (36.7)	
Caesarean section	67,387 (44.5)	946 (22.5)	8231 (32.4)	26,214 (40.8)	20,509 (52.2)	11,487 (63.3)	
V/C ratio	1.25	3.45	2.08	1.45	0.92	0.58	
Fetal gender							0.587
Male	76,126 (50.3)	2168 (51.5)	12,776 (50.2)	32,303 (50.3)	19,636 (50.0)	9243 (50.9)	
Female	67,213 (44.4)	1892 (45.0)	11,454 (45.0)	28,563 (44.5)	17,274 (43.9)	8030 (44.3)	
M/F ratio	1.13	1.13	1.08	1.08	1.07	1.12	
Anthropometric indicators of infants							
Birth height (cm)	49.85 ± 11.38	49.53 ± 12.35	49.74 ± 10.36	49.95 ± 11.52	49.87 ± 11.79	49.64 ± 11.16	0.003
Birth weight (g)	3265.91 ± 539.22	3109.50 ± 567.32	3221.73 ± 545.47	3289.32 ± 518.60	3287.23 ± 537.91	3246.66 ± 584.75	< 0.001

Note: P/M: primipara/multipara; V/C, vaginal deliveries/caesarean section; M/F, male/female

### Trends in childbearing age

As shown in **Table S1**, the average age at delivery increased from 27.1 years in 2010 to 28.7 years in 2021. However, the average age of multiparous women increased from 29.9 to 31.8 between 2010 and 2017, and after 2017, the average age decreased year by year to 30.9. Concurrently, the proportion of those who gave birth < 20 and 20–24 years old decreased by year. Specifically, the average annual percentage change (AAPC) of < 20 years old was − 13.4% (*95% CI*: −16.1% - −10.7%) between 2010 and 2021. Among individuals aged 20 to 24, the AAPC exhibited a downward trend during the same period from 2010 to 2021. The AAPC stood at −7.1% (*95% CI*: −9.9% to −4.3%). Notably, this downward trend was most pronounced from 2013 to 2017, with an APC of −15.0% (*95% CI*: −21.7% to −7.7%). Details are presented in Table 2.

**Table 2:** Trends of maternal age groups from 2010 to 2021 using join point regression analysis

Age group	Trend1	APC	Trend2	APC	Trend3	APC	AAPC
**Overall**							
**< 20 years old**	2010–2021	−13.4 (−16.1, −10.7)					−13.4 (−16.1,−10.7)
**20–24 years old**	2010–2013	−6.9 (−13.4, −0.0)	2013–2017	−15.0 (−21.7,−7.7)	2017–2021	1.3 (−5.1,8.2)	−7.1 (−9.9, −4.3)
**25–29 years old**	2010–2014	4.5 (−3.3, 13.0)	2014–2021	−1.5 (−4.1, 1.1)			0.6 (−2.1, 3.4)
**30–34 years old**	2010–2021	5.5 (4.6, 6.4)					5.5 (4.6, 6.4)
**≥ 35 years old**	2010–2014	−6.5 (−25.0, 16.6)	2014–2017	31.9 (−23.1, 126.2)	2017–2021	−10.2 (−24.4, 6.6)	1.2 (−10.8, 14.8)
**Primipara**							
**< 20 years old**	2010–2021	−12.7 (−14.4,11.1)					−12.7 (−14.4, −11.1)
**20–24 years old**	2010–2016	−10.2 (−12.2, −8.2)	2016–2021	−3.5 (−7.4,0.5)			−7.3 (−8.9, −5.5)
**25–29 years old**	2010–2016	5.2 (3.5,7.0)	2016–2021	−3.1 (−5.0, −1.2)			1.3 (0.3,2.4)
**30–34 years old**	2010–2013	9.3 (−1.2,20.8)	2013–2016	0.2 (−14.3,17.1)	2016–2021	10.8 (7.4,14.3)	7.4 (3.5,11.5)
**≥ 35 years old**	2010–2013	−5.2 (−21.8,14.8)	2013–2018	17.0 (6.2,28.9)	2018–2021	−0.8 (−11.7,11.5)	5.6 (0.1,11.4)
**Multipara**							
**< 20 years old**	2010–2013	14.1 (−25.0, 73.6)	2013–2018	−26.1 (−44.2, −2.0)	2018–2021	19.7 (−31.4, 109.0)	−5.1 (−19.3,11.6)
**20–24 years old**	2010–2013	1.0 (−8.9, 12.0)	2013–2017	−21.7 (−30.0, −12.5)	2017–2021	2.8 (−6.8, 13.5)	−7.3 (−11.2,−3.3)
**25–29 years old**	2010–2021	−1.8 (−3.0, −0.7)					−1.8 (−3.0, −0.7)
**30–34 years old**	2010–2021	3.6 (2.5, 4.7)					
**≥ 35 years old**	2010–2014	−6.5 (−16.6, 4.8)	2014–2017	17.7 (−11.2, 56.1)	2017–2021	−5.6 (−14.0, 3.7)	−0.1 (−6.5, 6.8)

APC, annual percentage change; AAPC, average annual percent change; CI, confidence interval

However, the proportions of women aged 30–34 and ≥ 35 years old both increased. As shown in Table 2, the population aged 30–34 exhibited a significant upward trend between 2010 and 2021 (*AAPC* = 5.5%, *95%CI*: 4.6% − 6.4%). From 2010 to 2021, women aged ≥ 35 years also exhibited an upward trend, with an AAPC of 1.2%, though this increase was not statistically significant. However, after stratification, advanced-age primiparas were found to have a significantly increasing trend, with an AAPC of 5.6% (95% CI: 0.1% − 11.4%).

Overall, the delay in childbearing age and the increasing proportion of advanced-age pregnant women are the main trends at present. These changes may bring higher risks of pregnancy adverse outcomes and the possibility of adverse outcomes.

### Relationship between maternal age and adverse outcomes

As shown in Table 1, both parity and the rate of cesarean section significantly increased with age (*P* < 0.001), and the proportions of primiparas and multiparas showed an inverted U-shaped relationship. For offspring, a U-shaped relationship between the male-to-female ratio and maternal age was observed in different age groups. In terms of anthropometric indices, infants born to mothers aged 25–29 had the highest birth weight and the longest birth length, which may suggest that childbirth by mothers in this age range could be more beneficial for offspring.

Table 3 presents the distribution of adverse outcomes across five maternal age groups, with separate analyses conducted for primiparous and multiparous women. Among all adverse pregnancy outcomes, the incidence of anemia was the highest, followed by PROM, GDM, and TD. The distribution of adverse outcomes was found to be relatively consistent between the two groups. Specifically, the incidences of GDM, placenta previa, polyhydramnios, PPH, and TD all increased with age. Anemia, LBW, preterm birth, PIH, placental abruption, and preeclampsia presented U - shaped distributions. Oligohydramnios and FGR showed relatively stable or declining trends across different age groups. Meanwhile, macrosomia and PROM displayed inverted U - shaped patterns among primiparas and in the overall population (Table 3).

**Table 3:** Distribution of adverse outcomes among different maternal age groups [*N* = 151,301 (%)]

Adverse outcomes	Total (%)	Age group					χ2	*P*
		< 20 years old (*n* = 4,206)	20–24 years old (*n* = 25,441)	25–29 years old (*n* = 64,197)	30–34 years old (*n* = 39,311)	≥ 35 years old (*n* = 18,146)		
**Overall**								
Anemia	35,550 (23.5)	1104 (26.2)	6179 (24.3)	14,157 (22.1)	9413 (23.9)	4697 (25.9)	163.01	< 0.001
GDM	20,884 (13.8)	143 (3.4)	1597 (6.3)	7540 (11.7)	7005 (17.8)	4599 (25.3)	4386.75	< 0.001
FGR	7597 (5.0)	252 (6.0)	1362 (5.4)	3413 (5.3)	1755 (4.5)	815 (4.5)	62.17	< 0.001
LBW	5888 (3.9)	196 (4.7)	904 (3.6)	1914 (3.0)	1688 (4.3)	1186 (6.5)	512.87	< 0.001
Macrosomia	8163 (5.4)	120 (2.9)	1090 (4.3)	3368 (5.2)	2441 (6.2)	1144 (6.3)	197.99	< 0.001
Oligohydramnios	9838 (6.5)	297 (7.1)	1744 (6.9)	4243 (6.6)	2442 (6.2)	1112 (6.1)	18.21	0.001
PIH	5672 (3.7)	156 (3.7)	791 (3.1)	2008 (3.1)	1559 (4.0)	1158 (6.4)	451.18	< 0.001
Placenta previa	4330 (2.9)	41 (1.0)	376 (1.5)	1415 (2.2)	1421 (3.6)	1077 (5.9)	1025.75	< 0.001
Placental abruption	2910 (1.9)	112 (2.7)	460 (1.8)	1133 (1.8)	750 (1.9)	455 (2.5)	55.40	< 0.001
Polyhydramnios	2657 (1.8)	34 (0.8)	314 (1.2)	1031 (1.6)	792 (2.0)	486 (2.7)	175.13	< 0.001
PPH	13,227 (8.7)	252 (6.0)	1687 (6.6)	5222 (8.1)	3841 (9.8)	2225 (12.3)	545.63	< 0.001
Preeclampsia	2035 (1.3)	65 (1.5)	399 (1.6)	649 (1.0)	500 (1.3)	422 (2.3)	197.90	< 0.001
Preterm birth	14,471 (9.6)	557 (13.2)	2457 (9.7)	5282 (8.2)	3809 (9.7)	2366 (13.0)	452.59	< 0.001
PROM	31,055 (20.5)	768 (18.3)	5192 (20.4)	13,873 (21.6)	7771 (19.8)	3451 (19.0)	98.85	< 0.001
TD	20,118 (13.3)	333 (7.9)	253 0(9.9)	8713 (13.6)	5808 (14.8)	2734 (15.1)	481.55	< 0.001
**Primipara**								
Anemia	19,592 (21.7)	961 (26.0)	4423 (23.4)	9740 (20.6)	3678 (21.8)	790 (22.8)	45.63	< 0.001
GDM	11,445 (12.7)	128 (3.5)	1270 (6.7)	5717 (12.1)	3310 (19.6)	1020 (29.5)	1170.35	< 0.001
FGR	5570 (6.2)	235 (6.4)	1185 (6.3)	2889 (6.1)	1059 (6.3)	202 (5.8)	0.91	0.634
LBW	2175 (2.4)	145 (3.9)	460 (2.4)	1011 (2.1)	437 (2.6)	122 (3.5)	58.93	< 0.001
Macrosomia	3929 (4.4)	99 (2.7)	729 (3.9)	2159 (4.6)	795 (4.7)	147 (4.2)	26.17	< 0.001
Oligohydramnios	6896 (7.6)	276 (7.5)	1478 (7.8)	3494 (7.4)	1370 (8.1)	278 (8.0)	0.90	0.636
PIH	3394 (3.8)	147 (4.0)	664 (3.5)	1555 (3.3)	779 (4.6)	249 (7.2)	118.53	< 0.001
Placenta previa	1867 (2.1)	32 (0.9)	254 (1.3)	880 (1.9)	518 (3.1)	183 (5.3)	205.94	< 0.001
Placental abruption	1700 (1.9)	100 (2.7)	327 (1.7)	829 (1.8)	343 (2.0)	101 (2.9)	36.44	< 0.001
Polyhydramnios	1318 (1.5)	31 (0.8)	225 (1.2)	690 (1.5)	301 (1.8)	71 (2.1)	18.30	< 0.001
PPH	7077 (7.8)	220 (6.0)	1269 (6.7)	3687 (7.8)	1498 (8.9)	403 (11.6)	87.99	< 0.001
Preeclampsia	1278 (1.4)	65 (1.8)	362 (1.9)	521 (1.1)	237 (1.4)	93 (2.7)	45.92	< 0.001
Preterm birth	7939 (8.8)	489 (13.2)	1792 (9.5)	3718 (7.9)	1502 (8.9)	438 (12.7)	168.28	< 0.001
PROM	21,311 (23.6)	715 (19.4)	4312 (22.8)	11,445 (24.2)	4056 (24.0)	783 (22.6)	41.46	< 0.001
TD	12,752 (14.1)	309 (8.4)	2077 (11.0)	6901 (14.6)	2855 (16.9)	610 (17.6)	136.79	< 0.001
**Multipara**								
Anemia	15,958 (26.1)	143 (27.8)	1756 (27.0)	4417 (26.0)	5735 (25.6)	3907 (26.6)	8.58	0.073
GDM	9439 (15.4)	15 (2.9)	327 (5.0)	1823 (10.7)	3695 (16.5)	3579 (24.4)	1802.94	< 0.001
FGR	2027 (3.3)	17 (3.3)	177 (2.7)	524 (3.1)	696 (3.1)	613 (4.2)	46.80	< 0.001
LBW	3713 (6.1)	51 (9.9)	444 (6.8)	903 (5.3)	1251 (5.6)	1064 (7.2)	81.33	< 0.001
Macrosomia	4234 (6.9)	21 (4.1)	361 (5.5)	1209 (7.1)	1646 (7.3)	997 (6.8)	33.10	< 0.001
Oligohydramnios	2942 (4.8)	21 (4.1)	266 (4.1)	749 (4.4)	1072 (4.8)	834 (5.7)	37.98	< 0.001
PIH	2278 (3.7)	9 (1.8)	127 (2.0)	453 (2.7)	780 (3.5)	909 (6.2)	367.43	< 0.001
Placenta previa	2463 (4.0)	9 (1.8)	122 (1.9)	535 (3.2)	903 (4.0)	894 (6.1)	279.35	< 0.001
Placental abruption	1210 (2.0)	12 (2.3)	133 (2.0)	304 (1.8)	407 (1.8)	354 (2.4)	20.71	< 0.001
Polyhydramnios	1339 (2.2)	3 (0.6)	89 (1.4)	341 (2.0)	491 (2.2)	415 (2.8)	56.98	< 0.001
PPH	6150 (10.1)	32 (6.2)	418 (6.4)	1535 (9.1)	2343 (10.4)	1822 (12.4)	215.60	< 0.001
Preeclampsia	757 (1.2)	0	37 (0.6)	128 (0.8)	263 (1.2)	329 (2.2)	183.98	< 0.001
Preterm birth	6532 (10.7)	68 (13.2)	665 (10.2)	1564 (9.2)	2307 (10.3)	1928 (13.1)	138.51	< 0.001
PROM	9744 (15.9)	53 (10.3)	880 (13.5)	2428 (14.3)	3715 (16.6)	2668 (18.2)	134.78	< 0.001
TD	7366 (12.1)	24 (4.7)	453 (7.0)	1812 (10.7)	2953 (13.2)	2124 (14.5)	322.32	< 0.001

Note: GDM, gestational diabetes mellitus; FGR: fetal growth restriction; PIH, pregnancy-induced hypertension; TD, thyroid dysfunction; LBW, low birth weight; PPH, postpartum hemorrhage; PROM, premature rupture of membranes

### The optimal childbearing age evaluated through join point regression analysis

To identify the optimal childbearing age, a join point regression analysis was performed after assessing the trends of adverse outcomes in this age range. Here, percent change per year of age (PCA) represented the percentage change in age for each segment.

The optimal childbearing age in the overall population.

**Fig. 2:**
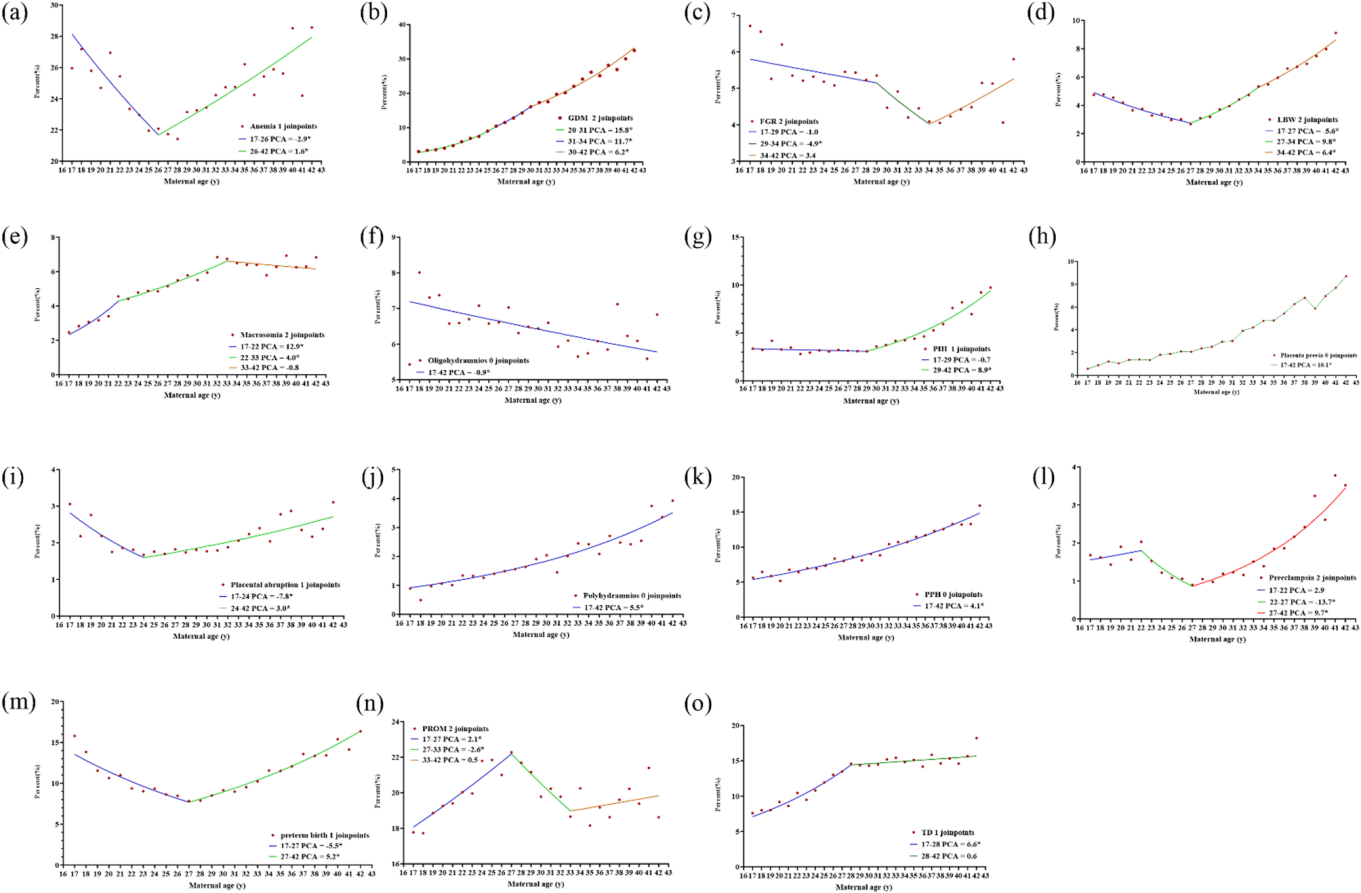
The trend of adverse outcomes incidence in relation to maternal age

As shown in Fig. 2, five adverse outcomes, namely, GDM, placenta previa, polyhydramnios, PPH and TD exhibited increasing trends with advanced age. Among the above-mentioned outcomes, placenta previa, polyhydramnios and PPH showed a more linear growth trend, while other trends appeared nonlinear. More specifically, the trend of GDM grew more sharply before age 26 (PCA 15.8%, *95% CI*: 13.4%−18.3%) than 26–30 (PCA 11.7%, *95% CI*: 8.0%−15.5%) and 30–42 (PCA 6.2, *95% CI*: 5.5%−6.9%). TD also showed a similar pattern, with a more rapidly increasing trend at ages 17–28 (PCA 6.6%, *95% CI*: 5.5%−7.8%) than ages 28–42 (PCA 0.6%, *95% CI*: −0.1%−1.3%). Six adverse outcomes, namely anemia, FGR, LBW, PIH, placental abruption, and preterm birth, demonstrate a trend of initially declining and then rising, and this pattern varies across different age groups. The age range within which anemia shows a downward trend is from 17 to 26, followed by an upward trend (PCA for 17–26 was − 2.9%, for 26–42 was 1.6%). FGR exhibits the most pronounced downward trend among women aged 29–34 (PCA − 4.9%). The next most significant decline occurs in the 17–29 (PCA − 1.0). Conversely, between the ages of 34–42, it shows an upward trend (PCA 3.4). Placenta abruption shows a downward trend before the age of 24 (PCA − 7.8%, *95% CI*: −12.0%- −3.4%), while PIH shows a downward trend before the age of 29 (PCA − 0.7%, *95% CI*: −2.2%−0.9%). Similar to LBW, preterm birth shows a downward trend before the age of 27 (PCA − 5.6% vs. −5.5%). Macrosomia, preeclampsia, and PROM show a three - stage trend, first rising, then declining, and finally rising again. The occurrence of macrosomia decreases during the age range of 33–42. For every one - year increase in age, the incidence decreases by 0.8%. Preeclampsia shows a downward trend among women aged 22–27 (PCA − 13.7%), while PROM shows a downward trend among those aged 27–33 (PCA − 2.6%). A marked downward trend was observed for the remaining oligohydramnios (PCA − 0.9%, *95% CI*: −1.3% - −0.4%).

**Fig. 3:**
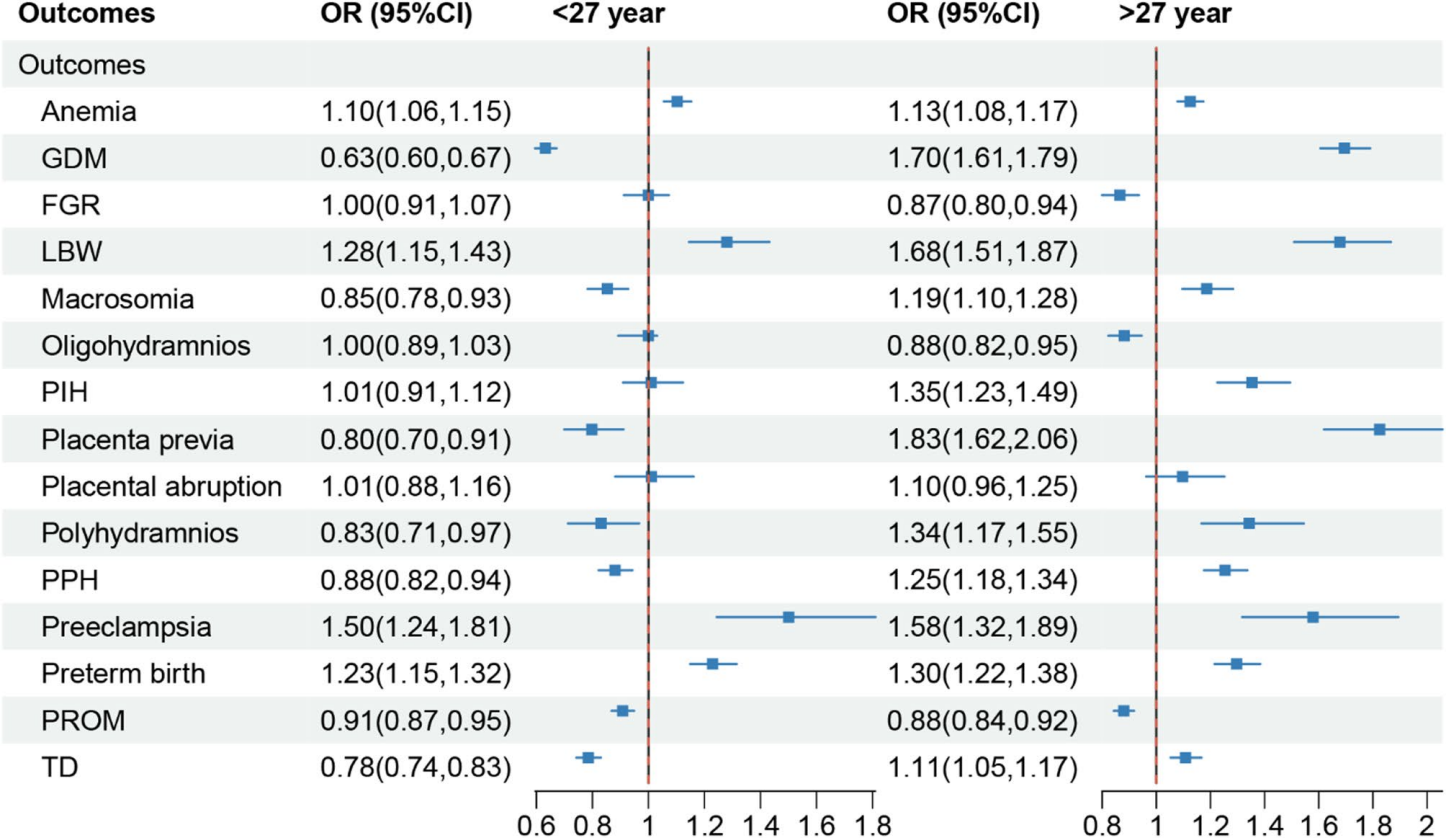
Logistic regression analysis of the association between age and adverse outcomes

We analyzed the intersection of all downward trends and found that the probabilities of the aforementioned adverse outcomes were consistently lower within the maternal age group of 22 to 33 years. By examining each junction, we ultimately determined that the optimal childbearing age, characterized by the lowest incidence of adverse outcomes, was 27 years. To further validate these findings, logistic regression analysis was also employed to examine the association between different maternal age groups and adverse outcomes. As illustrated in Fig. 3, compared with women aged 27 years, both those aged < 27 years and those aged > 27 years exhibited a significantly higher risk of anemia, LBW, preeclampsia, and preterm birth. Moreover, women aged > 27 years also faced a significantly increased risk of GDM, macrosomia, PIH, placenta previa, polyhydramnios, PPH, and TD. Collectively, these findings suggested that the optimal age range for childbearing was between 22 and 33 years, with 27 years being particularly recommended.

The optimal childbearing age for primiparas.

In this study, the average age of primiparous women was 26.9 years, which is lower than the overall population average of 28.5 years. Concurrently, to facilitate subsequent research on birth spacing, an analysis of the optimal childbearing age for primiparous women was also conducted. The analysis of the optimal age for primiparas using joinpoint analysis also shows that among the 15 adverse outcomes, there are three types of trends in total. GDM, placenta previa, polyhydramnios, TD, PPH, macrosomia, and PROM all show an upward trend. Anemia, PIH, preeclampsia, LBW, placental abruption, and preterm birth present a V-shaped pattern. However, FGR and oligohydramnios show no significant trends across different ages (**Supplementary Fig. 1**). Ultimately, the results indicate that the appropriate childbearing age for primiparas lies within the range of 22 to 26 years old, with 26 years old being the optimal age.

(a) The temporal trend in the prevalence of anemia; (b) The temporal trend in the prevalence of GDM; (c) The temporal trend in the prevalence of FGR; (d) The temporal trends in the prevalence of LBW; (e) The temporal trends in the prevalence of macrosomia; (f) The temporal trends in the prevalence of oligohydramnios; (g) The temporal trends in the prevalence of PIH; (h) The temporal trends in the prevalence of placenta previa; (i) The temporal trends in the prevalence of placental abruption; (j) The temporal trends in the prevalence of polyhydramnios; (k) The temporal trends in the prevalence of PPH; (l) The temporal trends in the prevalence of preeclampsia; (m) The temporal trends in the prevalence of preterm birth; (n) The temporal trends in the prevalence of PROM; (o) The temporal trends in the prevalence of TD.

The dots represented the actual data, and the lines represented the annual percentage change under the model.

The numbers of join points were selected by the optimal models from join point regression analysis.

The asterisk (*) indicated that the PCA was significantly different from zero at the α = 0.05 level. PCA, percent change per year of age; GDM, gestational diabetes mellitus; FGR: fetal growth restriction; PIH, pregnancy-induced hypertension; TD, thyroid dysfunction; LBW, low birth weight; PPH, postpartum hemorrhage; PROM, premature rupture of membrane.

The diamond represented the odds ratio (*OR*), and the line represented the 95% confidence interval (*CI*). GDM, gestational diabetes mellitus; FGR: fetal growth restriction; PIH, pregnancy-induced hypertension; TD, thyroid dysfunction; LBW, low birth weight; PPH, postpartum hemorrhage; PROM, premature rupture of membrane.

### The optimal birth spacing recommended by join point regression analysis

Given that the overall optimal childbearing age range in the aforementioned study was 22–33 years old, and individuals aged 20–34 accounted for over 92% in the age classification results, this age group was used for the analysis of the optimal birth spacing. A total of 8,222 pairs of primiparas and multiparas were successfully matched using identity card numbers. Among the primiparous women, those in the 20–34 age group accounted for 93.6%. Of the 7,693 subjects, 599 (7.8%) gave birth again within less than 2 years after their first delivery, 1,694 (22.0%) had a birth spacing of 2 years, 1,529 (19.9%) had a spacing of 3 years, 1,267 (16.5%) had a spacing of 4 years, 1,017 (13.2%) had a spacing of 5 years, and 1,587 (20.6%) had a spacing of more than 5 years. Among the aforementioned adverse outcomes, nine outcomes, including anemia, GDM, oligohydramnios, placenta previa, PPH, preeclampsia, preterm birth, PROM, and TD, exhibited significant differences across various birth spacing groups (Table 4).

**Table 4:** Distribution of adverse outcomes by maternal birth spacing [*N* = 7693 (%)]

Variables	All	< 2 years	2 years	3 years	4 years	5 years	> 5 years	χ^2^	*P*
**Number (%)**	7693 (100)	599 (7.8)	1694 (22.0)	1529 (19.9)	1267 (16.5)	1017 (13.2)	1587 (20.6)		
Anemia	1854 (24.1)	131 (21,9)	362 (21.4)	351 (23.0)	345 (27.2)	256 (25.2)	409 (25.8)	19.476	0.002
GDM	1279 (16.6)	74 (12.4)	226 (13.3)	245 (16)	208 (16.4)	192 (18.9)	334 (21)	47.605	< 0.001
FGR	174 (2.3)	8 (1.3)	36 (2.1)	28 (1.8)	34 (2.7)	30 (2.9)	38 (2.4)	7.073	0.215
LBW	150 (1.9)	15 (2.5)	28 (1.7)	24 (1.6)	26 (2.1)	19 (1.9)	38 (2.4)	4.646	0.461
Macrosomia	500 (6.5)	34 (5.7)	113 (6.7)	114 (7.5)	87 (6.9)	65 (6.4)	87 (5.5)	6.055	0.301
Oligohydramnios	286 (3.7)	29 (4.8)	55 (3.2)	53 (3.5)	39 (3.1)	31 (3.0)	79 (5.0)	13.196	0.022
PIH	179 (2.3)	9 (1.5)	33 (1.9)	39 (2.6)	28 (2.2)	20 (2.0)	50 (3.2)	8.593	0.126
Placenta previa	157 (2.0)	6 (1.0)	26 (1.5)	20 (1.3)	30 (2.4)	25 (2.5)	50 (3.2)	20.852	0.001
Placental abruption	158 (2.1)	16 (2.7)	32 (1.9)	33 (2.2)	25 (2.0)	20 (2.0)	32 (2.0)	1.537	0.909
Polyhydramnios	144 (1.9)	12 (2.0)	33 (1.9)	26 (1.7)	21 (1.7)	21 (2.1)	31 (2.0)	0.935	0.968
PPH	580 (7.5)	45 (7.5)	108 (6.4)	121 (7.9)	127 (10.0)	79 (7.8)	100 (6.3)	18.384	0.003
Preeclampsia	125 (1.6)	8 (1.3)	24 (1.4)	17 (1.1)	21 (1.7)	14 (1.4)	41 (2.6)	12.815	0.025
Preterm birth	556 (7.2)	47 (7.8)	106 (6.3)	93 (6.1)	94 (7.4)	66 (6.5)	150 (9.5)	18.315	0.003
PROM	1139 (14.8)	77 (12.9)	239 (14.1)	198 (12.9)	192 (15.2)	159 (15.6)	274 (17.3)	14.923	0.011
TD	1244 (16.2)	58 (9.7)	246 (14.5)	225 (14.7)	209 (16.5)	176 (17.3)	330 (20.8)	50.475	< 0.001

Note: GDM, gestational diabetes mellitus; FGR: fetal growth restriction; PIH, pregnancy-induced hypertension; TD, thyroid dysfunction; LBW, low birth weight; PPH, postpartum hemorrhage; PROM, premature rupture of membranes

**Fig. 4:**
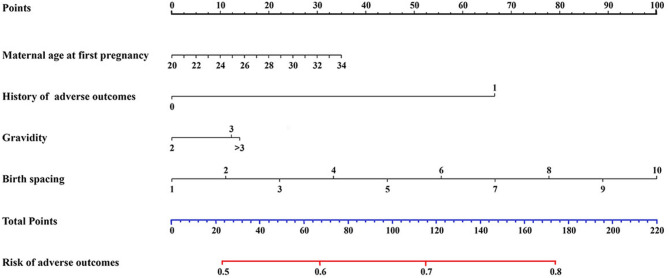
Nomogram to predict the risk of adverse outcomes in next pregnancy

We further conducted a nomogram analysis to evaluate birth spacing, maternal age at first pregnancy, gravidity, and history of adverse pregnancy outcomes. By comparing the score ranges and weights of different indicators on the nomogram, we can roughly determine which indicators have a greater impact on the outcomes. In this study, the data showed that the score range for birth spacing was relatively wide, highlighting the importance of studying the correlation between birth spacing and adverse outcomes (Fig. 4).

**Fig. 5:**
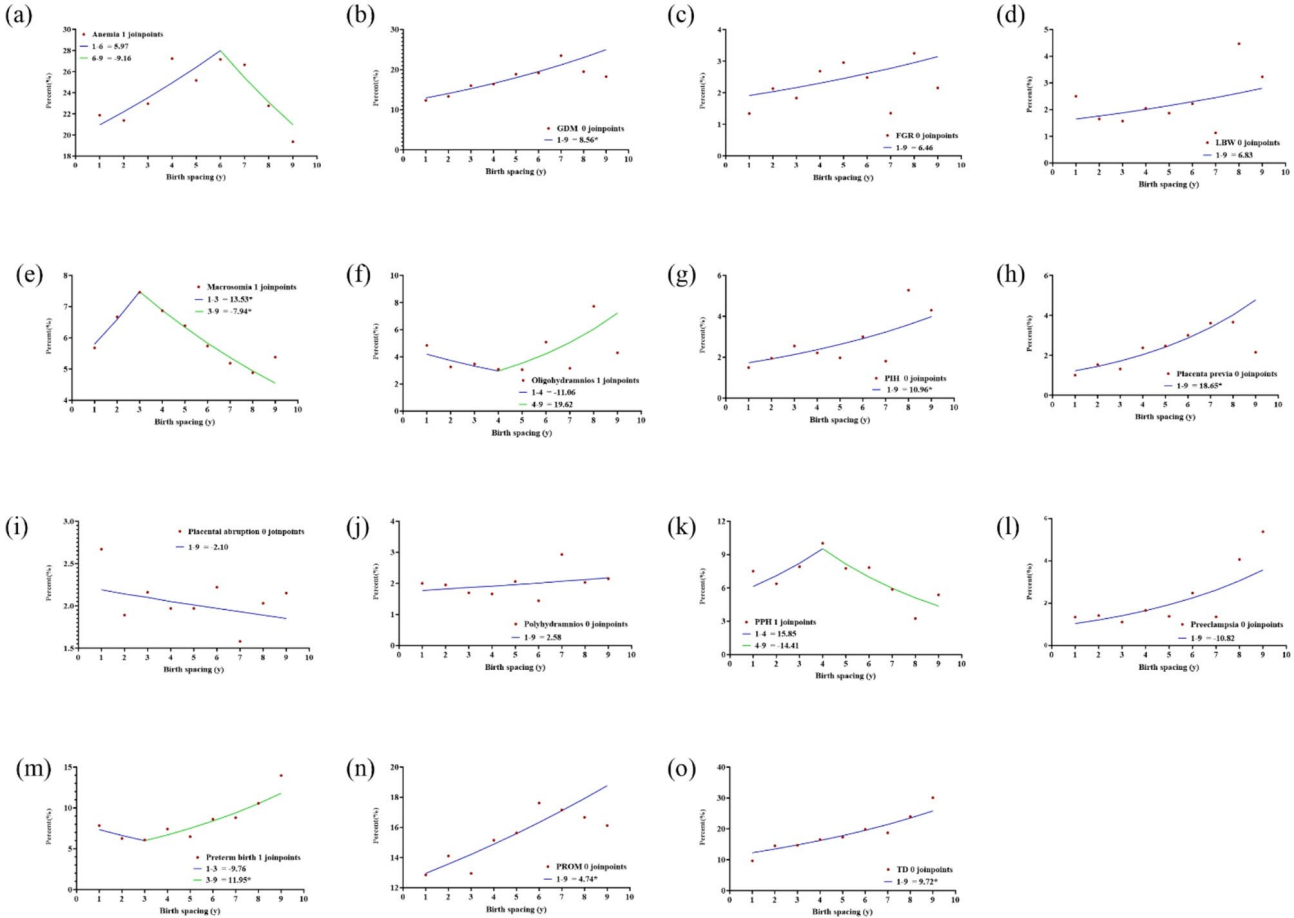
The trend of incidence for pregnancy complications at various birth spacing

Therefore, we subsequently used the joinpoint regression model to assess the trends of adverse outcomes across different birth spacing groups and determine the optimal childbearing birth spacing. Among all adverse outcomes, GDM, PIH, placenta previa, PROM and TD significantly increased with increasing birth spacing. Macrosomia first displayed a rising tendency before 3 years of birth spacing and then a falling tendency after 3 years. In contrast to macrosomia, preterm birth first showed a downward trend between 1 and 3 years and then a continuous rise after 3 years. Although the results were not statistically significant, other adverse outcomes all showed certain trends of change (Fig. 5). Furthermore, by analyzing the intersection of all declining trends, it was determined that the optimal birth spacing of 3 years occurred within the maternal age group of 20–34.

**Fig. 6:**
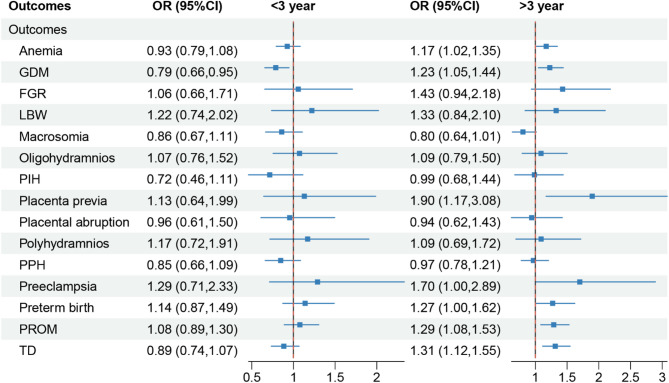
Logistic regression analysis of the association between birth spacing and adverse outcomes

Finally, we conducted a Logistic validation analysis, categorizing birth spacing into intervals of < 3 years, exactly 3 years, and > 3 years. Birth spacing > 3 years was significantly increased the risks of seven adverse outcomes: anemia, GDM, placenta previa, preeclampsia, preterm birth, PROM and TD (Fig. 6). At the same time, no significant protective effect was observed in the > 3-year group, though < 3 years of birth spacing showed a protective effect for GDM, and no significant risk effect was observed for other adverse outcomes. Further analysis revealed that < 2 years of birth spacing significantly increased the risk of oligohydramnios. Therefore, a mother’s birth spacing should be 2–3 years, preferably 3 years.

### Birth spacing under different conditions

Additionally, we explored the optimal birth spacing under different conditions. Table 5 summarizes the impact of the first pregnancy conditions of pregnant women (including mode of delivery, fetal sex, adverse pregnancy outcomes) on birth spacing and lists the optimal birth spacing under different conditions. The results showed that the fetal sex in the first pregnancy, mode of delivery, anemia, GDM, placenta previa, polyhydramnios, preterm delivery, TD, LBW, macrosomia, and PROM influenced birth spacing (Table 5). Using the Joinpoint analysis method to analyze the optimal birth spacing under different conditions, the results showed that the optimal birth spacing after a first male fetus was 5 years, while after a first female fetus it was 4 years. The best spacing after cesarean section was 5 years, and for natural birth it was 3 years. The spacing for a second birth should be 4 years for women who previously had GDM and 5 years for women who previously had anemia. To determine the recommended birth spacing for women with histories of placenta previa, polyhydramnios, TD, LBW and macrosomia, future studies with larger sample sizes are needed (Table 5).

**Table 5:** Actual and recommended birth spacing in different States

History of first delivery	State	Birth spacing	β	*P*	Recommended birth spacing
Cesarean section	No (Ref.)	3.49 ± 1.89	0.971^**^	< 0.001	3
	Yes	4.47 ± 1.89			5
First fetal gender	Female (Ref.)	3.81 ± 1.94	−0.110^*^	0.014	4
	Male	3.92 ± 1.96			5
Anemia	No (Ref.)	3.95 ± 1.97	−0.596^***^	< 0.001	4
	Yes	3.35 ± 1.77			5
GDM	No (Ref.)	3.89 ± 1.97	−0.377^***^	< 0.001	3
	Yes	3.51 ± 1.68			4
FGR	No (Ref.)	3.85 ± 1.96	0.067	0.481	NA
	Yes	3.91 ± 1.76			NA
Oligohydramnios	No (Ref.)	3.85 ± 1.95	0.068	0.465	NA
	Yes	3.91 ± 1.94			NA
PIH	No (Ref.)	3.85 ± 1.95	0.110	0.376	NA
	Yes	3.96 ± 1.99			NA
Placenta previa	No (Ref.)	3.84 ± 1.95	0.382^*^	0.021	NA
	Yes	4.23 ± 2.04			NA
Polyhydramnios	No (Ref.)	3.84 ± 1.95	0.380^*^	0.023	NA
	Yes	4.22 ± 2.04			NA
Preeclampsia	No (Ref.)	3.85 ± 1.95	−0.021	0.884	NA
	Yes	3.83 ± 1.80			NA
TD	No (Ref.)	3.94 ± 1.98	−0.991^***^	< 0.001	NA
	Yes	2.95 ± 1.33			NA
LBW	No (Ref.)	3.86 ± 1.95	−0.809^***^	< 0.001	NA
	Yes	3.05 ± 1.59			NA
Macrosomia	No (Ref.)	3.87 ± 1.96	−0.520^***^	< 0.001	NA
	Yes	3.35 ± 1.51			NA
Placental abruption	No (Ref.)	3.85 ± 1.95	−0.295	0.148	NA
	Yes	3.56 ± 1.82			NA
PPH	No (Ref.)	3.86 ± 1.96	−0.070	0.373	NA
	Yes	3.78 ± 1.80			NA
Preterm birth	No (Ref.)	3.84 ± 1.95	0.089	0.297	NA
	Yes	3.93 ± 1.97			NA
PROM	No (Ref.)	3.89 ± 1.97	−0.158^**^	0.002	4
	Yes	3.73 ± 1.89			4

Note: GDM, gestational diabetes mellitus; FGR: fetal growth restriction; PIH, pregnancy-induced hypertension; TD, thyroid dysfunction; LBW, low birth weight; PPH, postpartum hemorrhage; PROM, premature rupture of membranes, Ref: reference. * Linear regression results in p-value < 0.05; ** Linear regression results in *P*-value < 0.01; *** Linear regression results in *P*-value < 0.001

A nomogram was developed to predict the incidence of adverse outcomes. The model shape map was established based on factors including maternal age at first delivery, history of pregnancy adverse outcomes, gravidity, and birth spacing.

(a) The temporal trend in the prevalence of anemia; (b) The temporal trend in the prevalence of GDM; (c) The temporal trend in the prevalence of FGR; (d) The temporal trends in the prevalence of LBW; (e) The temporal trends in the prevalence of macrosomia; (f) The temporal trends in the prevalence of oligohydramnios; (g) The temporal trends in the prevalence of PIH; (h) The temporal trends in the prevalence of placenta previa; (i) The temporal trends in the prevalence of placental abruption; (j) The temporal trends in the prevalence of polyhydramnios; (k) The temporal trends in the prevalence of PPH; (l) The temporal trends in the prevalence of preeclampsia; (m) The temporal trends in the prevalence of preterm birth; (n) The temporal trends in the prevalence of PROM; (o) The temporal trends in the prevalence of TD. The dots represented the actual data, and the lines represented the annual percentage change under the model. The score in the picture represented the percent change per year of birth spacing. The asterisk (*) indicated that the score was significantly different from zero at the α = 0.05 level. GDM, gestational diabetes mellitus; FGR: fetal growth restriction; PIH, pregnancy-induced hypertension; TD, thyroid dysfunction; LBW, low birth weight; PPH, postpartum hemorrhage; PROM, premature rupture of membrane.

The diamond represents the odds ratio (*OR*), and the line represents the 95% confidence interval (*CI*).

GDM, gestational diabetes mellitus; FGR: fetal growth restriction; PIH, pregnancy-induced hypertension; TD, thyroid dysfunction; LBW, low birth weight; PPH, postpartum hemorrhage; PROM, premature rupture of membranes.

### Different optimal birth spacings recommended for varying maternal ages

Considering the interaction between age and birth spacing, we performed an analysis of birth spacing in different age groups. The results indicated that within the < 26-year age group, anemia, GDM, PIH, TD, and PPH exhibited increasing trends as birth spacing increased. Conversely, placenta previa and PROM demonstrated the lowest incidence rates at a birth spacing of 3 years. In the 26-year age group, birth spacing did not influence adverse outcomes. In the > 26-year age group, anemia, placenta previa, and TD showed the lowest incidence with a birth spacing of 3 years (**Table S2**). In addition, logistic regression analysis also confirmed that, the occurrence of adverse outcomes was not significantly influenced by birth spacing within the 26-year age group. Notably, intervals of ≤ 3 years between births yielded more favorable results compared to those observed in the < 26-year and > 26-year groups (**Table S3**).

## Discussion

Both maternal age and birth interval have been established as critical determinants of pregnancy outcomes. Our findings corroborate a significant trend of delayed childbearing in China, with a marked increase in the proportion of AMA primiparas. Notably, this analysis identifies 27 years as the optimal maternal age and 3 years as the ideal birth interval.

From 2010 to 2021, the trend of advanced maternal age (≥ 35 years) has shown a significant increase, with an average annual percentage change (AAPC) of 1.2% in the overall population and 5.6% among primiparous women. This highlights the phenomenon of delayed childbirth. Despite extensive research on delayed motherhood, limited studies have concurrently examined the optimal age for childbearing and birth spacing. Studies have shown that higher education levels, professional occupations, and changes in childbearing policies have all contributed to the increasing trend of advanced maternal age [34]. However, delayed childbearing is associated with an increased risk of maternal pregnancy complications and adverse perinatal outcomes. Therefore, it is necessary to inform women that delayed childbearing and advanced maternal age may affect conception, pregnancy, and perinatal outcomes.

Consequently, we employed joinpoint regression analysis and logistic regression to address this gap. The findings indicated that the age range of 22 to 33 years was relatively optimal for childbirth, with a particular emphasis on the age of 27. Firstly, based on the results of this study, the average age of childbearing for the total population of 151,301 was 28.5 years, falling within the age range of 22 to 33 years. Secondly, from a fertility perspective previous, studies indicated that the optimal maternal age range for childbirth, associated with lower risks, was between 20 and 30 years [20]. From the perspective of fertility, a North American preconception cohort study observed that fertility declines almost monotonically with increasing female age, with a notable exception between the ages of 28 and 33, during which fertility remains relatively stable [21]. The levels of Anti-Mullerian Hormone (AMH) are closely related to fertility. Studies have shown that AMH levels steadily increase from birth, peak at the age of 9, slightly decline during puberty, peak again around the age of 25, and then decline more rapidly after the mid-30s until they become undetectable at menopause [36]. Moreover, in naturally conceived women, low maternal AMH levels in the first trimester are predictive of PIH, and abnormal AMH levels are associated with preterm birth and intrahepatic cholestasis of pregnancy [37–39]. Lastly, regarding adverse outcomes, the risk associated with maternal age shows a bimodal distribution. A retrospective cohort study of 43,537 nulliparous women under the age of 25 showed that compared with women aged 20 to 24.5 years, the younger the age, the higher the risk of preterm birth and anemia. The risk of anemia in older adolescents (16-19.9 years) increased by 1.15 times, and the risk of preterm delivery increased by 1.16 times. For younger adolescents (≤ 15.9 years), the risk of anemia during pregnancy increased by 1.25 times, and the risk of preterm birth increased by 1.36 times [40]. Meanwhile, older pregnant women have significantly higher risk of adverse outcomes. A population-based retrospective study showed a significant increase in maternal and fetal adverse outcomes such as GDM, gestational hypertension, cesarean section, preterm birth, LBW, macrosomia, and stillbirth between the ages of 30–34 and especially over 35 years of age [41]. The risk in the adolescent group is mainly due to the immaturity of their reproductive system [42], while the risk of the elderly group is suggested to be associated with the shortening of telomeres and increased oxidative stress with age [43], which may reduce the quality of oocytes [44], increase uterine and placental dysfunction, and thereby increase adverse outcomes [45, 46]. These findings support our results to some extent, which suggest that 27 years old is the optimal age for childbearing. At this stage, the body’s hormonal status is more conducive to fertility and reduces the risk of complications.

We simultaneously employed joinpoint regression analysis to examine birth spacing. By identifying the intersection of birth spacing where the trends of adverse pregnancy outcomes declined, we concluded that the appropriate birth spacing is 2 to 3 years. After determining the junctions, we determined that the optimal birth spacing is 3 years. A retrospective cross-sectional study involving 456,889 parous women who delivered singleton infants revealed that compared to those conceiving 18–23 months after a previous birth, women with interpregnancy intervals of 5 months or less had 1.72-fold and 1.30-fold higher risks of PROM and anemia, respectively [47]. Furthermore, compared to women with interpregnancy intervals of 18 to 23 months, those with intervals exceeding 59 months exhibited a 1.83-fold increased risk of preeclampsia and a 1.80-fold increased risk of eclampsia [47].

A systematic review and dose-response meta-analysis revealed in the general population, compared with an interpregnancy interval of 18–23 months, extreme intervals (< 6 months and ≥ 60 months) were associated with an increased risk of adverse outcomes, including preterm birth, small for gestational age, LBW, fetal death, birth defects, early neonatal death, and PROM (OR range: 1.08–1.56). The dose-response analyses further confirmed these J-shaped relationships [21]. Differences in optimal birth spacing may be explained by the age composition of the study population. The research included the 20–34 age group for the analysis of delivery intervals, whereas other studies encompassed delivery populations across all age groups.

With short birth spacing, the mother usually does not have enough time to recover from the physiological stress of a previous pregnancy, and consecutive pregnancies and lactation periods would lead to depletion of maternal nutrient reserves, such as the folate hypothesis, which increases the risks of adverse outcomes [48]. The specific potential mechanism between a long birth spacing and adverse neonatal outcomes is still not clear. It may be associated with the hypothesis of physiological regression, which suggests that the physiological reproductive capacity of women gradually declines with time soon after childbirth and returns to the status of first-time pregnant women [49]. In addition, long intervals may be associated with infertility and metabolic diseases, which need further research to be ruled out.

Maternal age, mode of delivery, and adverse outcomes may potentially influence the decision regarding birth spacing [50, 51]. We found that cesarean section and a first male baby significantly increased both the actual and recommended birth spacings, with an actual cesarean birth interval of 4.47 years, which was close to our calculated optimal birth spacing of 5 years. The research conducted by Jiaming Rao et al. indicated that the recommended interval for attempting trial delivery following a cesarean section was between 2 and 10 years [52]. Uterine rupture and abnormal placentation are the most common adverse outcomes in pregnancies following cesarean section. Short interpregnancy intervals are associated with an increased risk of uterine rupture in subsequent pregnancies [53]. Additionally, our data indicated that the optimal birth spacing for having a second child following the birth of a male firstborn was five years, whereas the observed average spacing was 3.92 years. Studies have shown that the average birth weight of male newborns is significantly higher than that of female newborns, which poses a potential risk of macrosomia. Moreover, male fetuses are a risk factor for cesarean section and can significantly increase the rate of cesarean delivery, both of which can lead to a delay in birth spacing [54]. Furthermore, for women with a history of GDM, PROM, or anemia, the recommended birth spacing exceeds current practice. The findings also suggested that the influence of extended birth spacing on the incidence of placenta previa diminished with increasing maternal age. Further research is necessary to elucidate the underlying mechanisms and provide more specific details.

This study has the following limitations. First, there is a limitation in sample representativeness. The samples of this study were sourced from 151,301 birth records registered in multiple tertiary hospitals in Ningbo City, eastern China, from 2010 to 2021. Although the sample size is statistically significant at the regional level, it has obvious deficiencies in representativeness at the national level. As a relatively economically developed city along the eastern coast, Ningbo may have systematic differences in population structure, medical resource allocation, and socio-cultural background compared with other regions. In addition, tertiary hospitals handle a higher proportion of high-risk pregnancy cases compared to primary healthcare institutions. The above factors may lead to deviations between indicators such as the age distribution of parturient and pregnancy outcomes and the national overall situation. Moreover, in the analysis of birth spacing, differences were observed between the distribution of matched multiparous women and the total multiparous population, which further limits the generalizability of the data. Therefore, although this study reveals the trends of changes in parturient age and their correlations within specific regions and medical institutions, due to the limited sample coverage, the research results cannot be directly generalized to the whole country.

Second, there is detection bias caused by the change in diagnostic criteria. The sample recruitment period of this study spanned more than 10 years (2010–2021). During this period, the clinical diagnostic protocol for GDM transitioned from a two-step method to a one-step method with a higher detection rate. This change in diagnostic criteria may introduce potential detection bias in the research results of different years, affecting the accurate analysis and interpretation of GDM-related data. In the follow-up, the investigation time will be further extended to ensure that all diseases are within the same diagnostic timeframe and reduce bias.

Third, there is a lack of data integrity. This study failed to collect data on parturients’ gestational body mass index (BMI), nutritional intake, breastfeeding practices, economic status, childbirth conditions (such as the duration of labor and fetal status), and socio-cultural factors. The absence of these data has caused a certain degree of confounding bias. In the follow-up, more complete data will be collected for matching or stratified analysis to reduce bias and further improve the understanding of childbearing age and birth spacing under different conditions, such as the optimal birth spacing under different feeding patterns.

Despite the above limitations, the total sample size of this study reached 151,301 cases. The large sample size enabled us to conduct reliable and meaningful statistical comparisons among different parturient age groups and birth spacing. We not only analyzed the impacts of parturient age and birth spacing on adverse outcomes but also explored the optimal parturient age and birth spacing. Moreover, by comprehensively considering women’s relatively best reproductive status and infants’ health, we studied the ideal childbearing plans under different conditions, providing an important basis for research on maternal and infant health in the social context of delayed childbearing.

## Conclusion

In conclusion, this study offers substantial evidence that the average age at delivery in China has exhibited an upward trajectory. The 20–34 age group remains the most active in childbearing, accounting for the majority of deliveries. Leveraging a multicenter research design, we determined that the optimal childbearing age is 27 years for the general population and 26 years for primiparous women. A birth spacing of three years is advisable, though this interval may vary across different demographic subgroups.

By emphasizing the optimal childbearing age and spacing, clinicians can better provide personalized fertility advice to women of childbearing age. For example, for couples planning to have children, doctors can offer professional recommendations on the best timing and spacing of childbirth based on their age and reproductive history, thereby reducing the risk of adverse pregnancy outcomes. Moreover, these research findings can also inform the development of public health policies. For instance, through community outreach and educational campaigns, public awareness of reproductive health can be enhanced. Future research should focus on exploring the optimal birth spacing and maternal age in different populations under more specific conditions, as well as investigating the underlying mechanisms.

## Supplementary Information

Below is the link to the electronic supplementary material.

Supplementary Material 1:Figure S1: The incidence trend of adverse outcomes at different primipara’s birth (a) The temporal trend in the prevalence of anemia (b) The temporal trend in the prevalence of GDM(c) The temporal trend in the prevalence of FGR (d) The temporal trends in the prevalence of LBW (e) The temporal trends in the prevalence of macrosomia (f) The temporal trends in the prevalence of oligohydramnios (g) The temporal trends in the prevalence of PIH (h) The temporal trends in the prevalence of placenta previa (i) The temporal trends in the prevalence of placental abruption (j) The temporal trends in the prevalence of polyhydramnios (k) The temporal trends in the prevalence of PPH (l) The temporal trends in the prevalence of preeclampsia (m) The temporal trends in the prevalence of preterm birth (n) The temporal trends in the prevalence of PROM (o) The temporal trends in the prevalence of TD The dots represent the actual data, and the lines represent the percent change per year of birth spacing under the model. GDM, gestational diabetes mellitus; FGR: fetal growth restriction; PIH, pregnancy- induced hypertension; TD, thyroid dysfunction; LBW, low birth weight; PPH, postpartum hemorrhage; PROM, premature rupture of membranes.

Supplementary Material 2

Supplementary Material 3

Supplementary Material 4

## Data Availability

No datasets were generated or analysed during the current study.

## Abbreviations

AMA: advanced maternal age
GDM: gestational diabetes mellitus
TD: thyroid dysfunction
PROM: premature rupture of membranes
PIH: pregnancy-induced hypertension
PPH: postpartum hemorrhage
LBW: low birth weight
FGR: fetal growth restriction
OR: odds ratio
CI: confidence interval
AMH: Anti-Mullerian hormone
PCA: percent change per year of age
AAPC: average annual percentage change
APC: annual percentage change
OGTT: oral glucose tolerance test
PCOS: polycystic ovarian syndrome
ART: assisted reproductive technology
