# Supplementary figures and images for: The optimal childbearing age and birth spacing in china: a multicenter retrospective cohort study
Source: BMC Public Health. 2025 Aug 30;25:2983. doi: 10.1186/s12889-025-24466-6 (PMC12398996; doi:10.1186/s12889-025-24466-6)

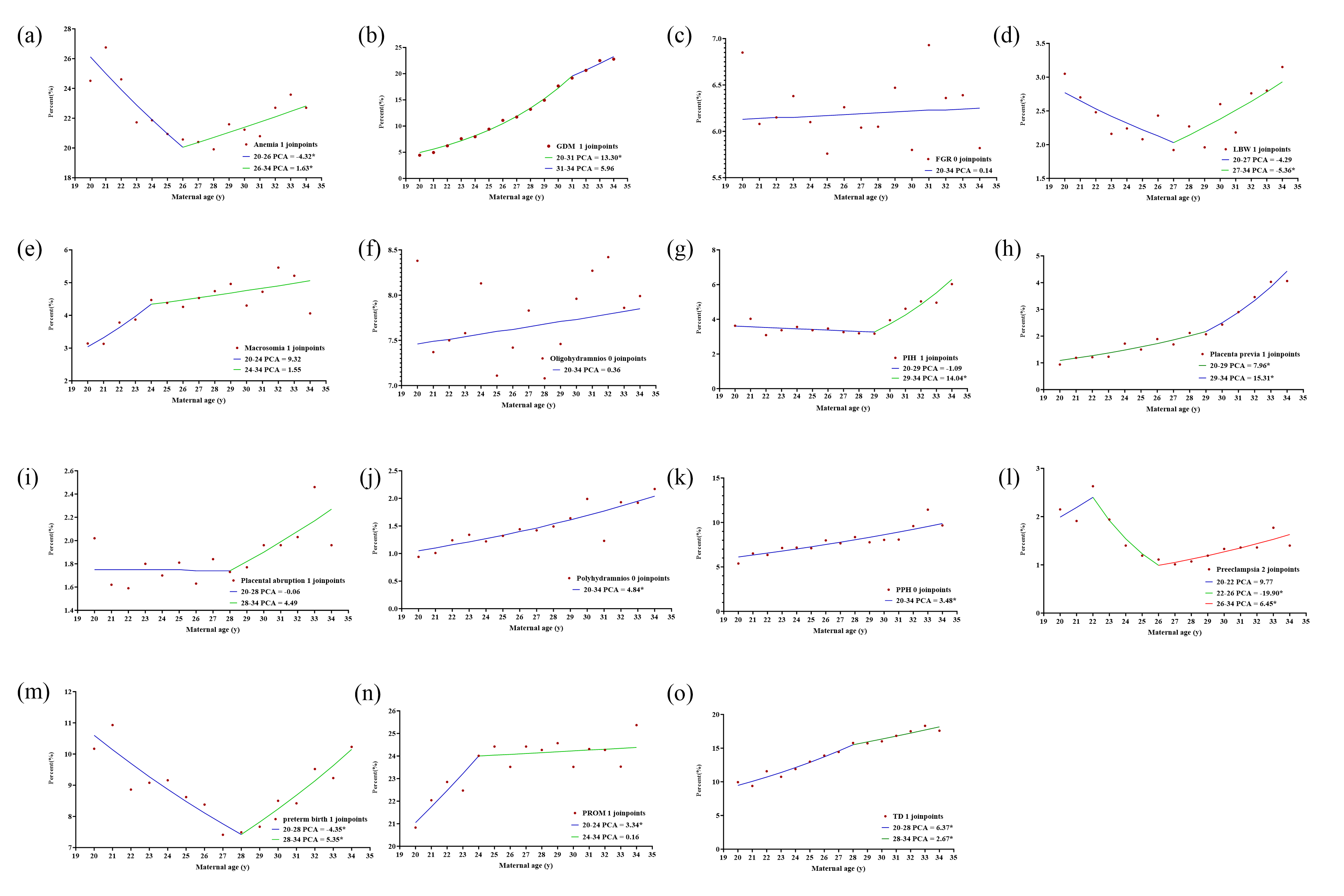

Supplement: Supplementary file 1 — Supplementary Material 1:Figure S1: The incidence trend of adverse outcomes at different primipara’s birth (a) The temporal trend in the prevalence of anemia (b) The temporal trend in the prevalence of GDM(c) The temporal trend in the prevalence of FGR (d) The temporal trends in the prevalence of LBW (e) The temporal trends in the prevalence of macrosomia (f) The temporal trends in the prevalence of oligohydramnios (g) The temporal trends in the prevalence of PIH (h) The temporal trends in the prevalence of placenta previa (i) The temporal trends in the prevalence of placental abruption (j) The temporal trends in the prevalence of polyhydramnios (k) The temporal trends in the prevalence of PPH (l) The temporal trends in the prevalence of preeclampsia (m) The temporal trends in the prevalence of preterm birth (n) The temporal trends in the prevalence of PROM (o) The temporal trends in the prevalence of TD The dots represent the actual data, and the lines represent the percent change per year of birth spacing under the model. GDM, gestational diabetes mellitus; FGR: fetal growth restriction; PIH, pregnancy- induced hypertension; TD, thyroid dysfunction; LBW, low birth weight; PPH, postpartum hemorrhage; PROM, premature rupture of membranes. [file 12889_2025_24466_MOESM1_ESM.tif]
