# Supplementary material for: The optimal childbearing age and birth spacing in china: a multicenter retrospective cohort study
Source: BMC Public Health. 2025 Aug 30;25:2983. doi: 10.1186/s12889-025-24466-6 (PMC12398996; doi:10.1186/s12889-025-24466-6)
Supplement: Supplementary file 2 — Supplementary Material 2 [file 12889_2025_24466_MOESM2_ESM.docx]

| **Year** | **All** | **Mean age** | **Primipara mean age** | **Multipara**  **mean age** | **<20 years old** | **20-24 years old** | **25-29years old** | **30-34 years old** | **≥35 years old** |
| --- | --- | --- | --- | --- | --- | --- | --- | --- | --- |
| **2010** | 9186 (6.1) | 27.1±5.3 | 25.4±4.3 | 29.9±5.4 | 496 (5.4) | 2585 (28.1) | 3416 (37.2) | 1751 (19.1) | 938 (10.2) |
| **2011** | 10519 (7.0) | 27.3±5.2 | 25.9±4.3 | 30.1±5.6 | 544 (5.2) | 2719 (25.8) | 4264 (40.5) | 1942 (18.5) | 1050 (10.0) |
| **2012** | 10537 (7.0) | 27.4±4.9 | 26.2±4.1 | 29.7±5.4 | 461 (4.4) | 248 2(23.6) | 4473 (42.5) | 2207 (20.9) | 914 (8.7) |
| **2013** | 11207 (7.4) | 27.4±4.9 | 26.2±4.0 | 29.7±5.4 | 491 (4.4) | 2547 (22.7) | 4804 (42.9) | 2420 (21.6) | 945 (8.4) |
| **2014** | 15229 (10.1) | 27.7±4.6 | 26.5±3.8 | 30.0±5.1 | 502 (3.3) | 2881 (18.9) | 7140 (46.9) | 3451 (22.7) | 1255 (8.2) |
| **2015** | 13836 (9.1) | 28.1±4.8 | 26.6±4.0 | 30.3±5.0 | 442 (3.2) | 2366 (17.1) | 6255 (45.2) | 3345 (24.2) | 1428 (10.3) |
| **2016** | 13229 (8.7) | 29.0±4.7 | 27.2±3.8 | 31.2±4.7 | 247 (1.9) | 1701 (12.9) | 5996 (45.3) | 3595 (27.2) | 1690 (12.8) |
| **2017** | 12752 (8.4) | 29.6±5.0 | 27.3±3.9 | 31.8±4.9 | 213 (1.7) | 1527 (12.0) | 5163 (40.5) | 3617 (28.4) | 2232 (17.5) |
| **2018** | 11017 (7.3) | 29.6±4.9 | 27.6±4.0 | 31.6±4.9 | 173 (1.6) | 1314 (11.9) | 4404 (40.0) | 3218 (29.2) | 1908 (17.3) |
| **2019** | 17146 (11.3) | 29.3±4.7 | 27.6±3.9 | 31.3±4.7 | 227 (1.3) | 2075 (12.1) | 7156 (41.7) | 5232 (30.5) | 2456 (14.3) |
| **2020** | 15627 (10.3) | 29.6±4.7 | 27.8±4.0 | 31.5±4.7 | 221 (1.4) | 1851 (11.8) | 6070 (38.8) | 5128 (32.8) | 2357 (15.1) |
| **2021** | 11016 (7.3) | 28.7±4.3 | 28.1±3.9 | 30.9±4.8 | 189 (1.7) | 1393 (12.6) | 5056 (45.9) | 3405 (30.9) | 973 (8.8) |
| **Total** | 151301 (100) | 28.5±4.9 | 26.9±4.1 | 30.8±5.0 | 4206 (2.8) | 25441 (16.8) | 64197 (42.4) | 39311 (26.0) | 18146 (12.0) |

**Supplementary table 1 Proportion of different maternal age groups from 2010 to 2021**
