# Supplementary material for: The optimal childbearing age and birth spacing in china: a multicenter retrospective cohort study
Source: BMC Public Health. 2025 Aug 30;25:2983. doi: 10.1186/s12889-025-24466-6 (PMC12398996; doi:10.1186/s12889-025-24466-6)
Supplement: Supplementary file 3 — Supplementary Material 3 [file 12889_2025_24466_MOESM3_ESM.docx]

**Supplementary table 2 The analysis of adverse outcomes incidence in various age groups with different birth spacing**

|  | **<26 years old** | | | | | **26 years old** | | | | | **>26 years old** | | | | |
| --- | --- | --- | --- | --- | --- | --- | --- | --- | --- | --- | --- | --- | --- | --- | --- |
|  | **<3** | **3** | **>3** | ***χ^2^*** | ***P*** | **<3** | **3** | **>3** | ***χ^2^*** | ***P*** | **<3** | **3** | **>3** | ***χ^2^*** | ***P*** |
| **Number** | 1156(34.8) | 648(19.5) | 1515(45.6) |  |  | 237(26.3) | 197(21.9) | 467(51.8) |  |  | 900(25.9) | 684(19.7) | 1889(54.4) |  |  |
| Anemia | 260(22.5) | 178(27.5) | 427(28.2) | 17.130 | <0.001 | 52(21.9) | 39(19.8) | 109(23.3) | 1.020 | 0.601 | 181(20.1) | 134(19.6) | 474(25.1) | 12.476 | 0.002 |
| GDM | 89(7.7) | 77(11.9) | 243(16) | 42.362 | <0.001 | 32(13.5) | 33(16.8) | 70(15) | 0.892 | 0.64 | 179(19.9) | 135(19.7) | 421(22.3) | 3.140 | 0.208 |
| FGR | 20(1.7) | 14(2.2) | 42(2.8) | 3.243 | 0.198 | 6(2.5) | 2(1.0) | 10(2.1) | 1.366 | 0.505 | 18(2.0) | 12(1.8) | 50(2.6) | 2.275 | 0.321 |
| LBW | 23(2.0) | 8(1.2) | 35(2.3) | 2.694 | 0.26 | 3(1.3) | 3(1.5) | 13(2.8) | 2.174 | 0.337 | 17(1.9) | 13(1.9) | 35(1.9) | 0.008 | 0.996 |
| Macrosomia | 73(6.3) | 45(6.9) | 105(6.9) | 0.462 | 0.794 | 16(6.8) | 15(7.6) | 29(6.2) | 0.444 | 0.801 | 58(6.4) | 54(7.9) | 105(5.6) | 4.759 | 0.093 |
| Oligohydramnios | 45(3.9) | 24(3.7) | 48(3.2) | 1.087 | 0.581 | 6(2.5) | 9(4.6) | 22(4.7) | 2.033 | 0.362 | 33(3.7) | 20(2.9) | 79(4.2) | 2.234 | 0.327 |
| PIH | 19(1.6) | 16(2.5) | 50(3.3) | 7.239 | 0.027 | 6(2.5) | 6(3.0) | 15(3.2) | 0.252 | 0.881 | 17(1.9) | 17(2.5) | 33(1.7) | 1.458 | 0.482 |
| Placenta previa | 13(1.1) | 3(0.5) | 31(2.0) | 9.229 | 0.010 | 2(0.8) | 6(3.0) | 9(1.9) | 2.826 | 0.243 | 17(1.9) | 11(1.6) | 65(3.4) | 9.374 | 0.009 |
| Placental abruption | 30(2.6) | 10(1.5) | 23(1.5) | 4.629 | 0.099 | 5(2.1) | 5(2.5) | 9(1.9) | 0.250 | 0.882 | 13(1.4) | 18(2.6) | 45(2.4) | 3.286 | 0.193 |
| Polyhydramnios | 17(1.5) | 9(1.4) | 28(1.8) | 0.869 | 0.647 | 6(2.5) | 5(2.5) | 8(1.7) | 0.735 | 0.692 | 22(2.4) | 12(1.8) | 37(2.0) | 1.076 | 0.584 |
| PPH | 67(5.8) | 56(8.6) | 119(7.9) | 6.285 | 0.043 | 19(8.0) | 16(8.1) | 33(7.1) | 0.323 | 0.851 | 67(7.4) | 49(7.2) | 154(8.2) | 0.869 | 0.648 |
| Preeclampsia | 12(1.0) | 8(1.2) | 33(2.2) | 6.097 | 0.047 | 2(0.8) | 1(0.5) | 11(2.4) | 4.152 | 0.125 | 18(2.0) | 8(1.2) | 32(1.7) | 1.647 | 0.439 |
| Preterm birth | 76(6.6) | 35(5.4) | 112(7.4) | 2.931 | 0.231 | 7(3.0) | 13(6.6) | 29(6.2) | 3.902 | 0.142 | 70(7.8) | 45(6.6) | 169(8.9) | 4.007 | 0.135 |
| PROM | 148(12.8) | 70(10.8) | 247(16.3) | 13.548 | 0.001 | 34(14.3) | 29(14.7) | 73(15.6) | 0.230 | 0.891 | 134(14.9) | 99(14.5) | 305(16.1) | 1.409 | 0.494 |
| TD | 143(12.4) | 102(15.7) | 273(18) | 15.900 | <0.001 | 34(14.3) | 32(16.2) | 91(19.5) | 3.131 | 0.209 | 127(14.1) | 91(13.3) | 351(18.6) | 14.787 | 0.001 |

Note: GDM: gestational diabetes mellitus; FGR: fetal growth restriction; PIH: pregnancy-induced hypertension; TD: thyroid dysfunction; LBW: low birth weight; PPH: postpartum hemorrhage; PROM: premature rupture of membranes.
