# Supplementary material for: The optimal childbearing age and birth spacing in china: a multicenter retrospective cohort study
Source: BMC Public Health. 2025 Aug 30;25:2983. doi: 10.1186/s12889-025-24466-6 (PMC12398996; doi:10.1186/s12889-025-24466-6)
Supplement: Supplementary file 4 — Supplementary Material 4 [file 12889_2025_24466_MOESM4_ESM.docx]

**Supplementary table 3 Logistic regression analysis of birth spacing associated with adverse pregnancy outcomes in various age group**

|  | **<26 years old** | | **26 years old** | | **>26 years old** | |
| --- | --- | --- | --- | --- | --- | --- |
|  | **<3** | **>3** | **<3** | **>3** | **<3** | **>3** |
| Anemia | 0.77(0.61,  0.96) | 1.04(0.84,  1.27) | 1.14(0.71,  1.82) | 1.23(0.82,  1.86) | 1.03(0.81,  1.33) | 1.38(1.11,  1.71) |
| GDM | 0.62(0.45,  0.85) | 1.42(1.08,  1.86) | 0.78(0.46,  1.32) | 0.88(0.56,  1.38) | 1.01(0.79,  1.30) | 1.17(0.94,  1.45) |
| FGR | 0.80(0.40,  1.59) | 1.29(0.70,  2.38) | 2.53(0.51,  12.69) | 2.13(0.46,  9.83) | 1.14(0.55,  2.39) | 1.52(0.81,  2.88) |
| LBW | 1.62(0.72,  3.65) | 1.89(0.87,  4.10) | 0.83(0.17,  4.15) | 1.85(0.52,  6.57) | 0.99(0.48,  2.06) | 0.97(0.51,  1.85) |
| Macrosomia | 0.90(0.62,  1.33) | 0.99(0.70,  1.43) | 0.88(0.42,  1.83) | 0.80(0.42,  1.53) | 0.80(0.55,  1.18) | 0.69(0.49,  0.97) |
| Oligohydramnios | 1.05(0.64,  1.75) | 0.85(0.52,  1.40) | 0.54(0.19,  1.55) | 1.03(0.47,  2.29) | 1.26(0.72,  2.22) | 1.45(0.88,  2.39) |
| PIH | 0.66(0.34,  1.29) | 1.35(0.76,  2.39) | 0.83(0.26,  2.61) | 1.06(0.40,  2.76) | 0.76(0.38,  1.49) | 0.70(0.39,  1.26) |
| Placenta previa | 2.45(0.69,  8.61) | 4.49(1.37,  14.74) | 0.27(0.05,  1.36) | 0.63(0.22,  1.78) | 1.18(0.55,  2.53) | 2.18(1.14,  4.16) |
| Placental abruption | 1.70(0.83,  3.50) | 0.98(0.47,  2.08) | 0.83(0.24,  2.90) | 0.76(0.25,  2.28) | 0.54(0.26,  1.12) | 0.90(0.52,  1.57) |
| Polyhydramnios | 1.06(0.47,  2.39) | 1.34(0.63,  2.85) | 0.99(0.30,  3.32) | 0.67(0.22,  2.07) | 1.40(0.69,  2.86) | 1.12(0.58,  2.16) |
| PPH | 0.65(0.45,  0.94) | 0.90(0.65,  1.26) | 0.99(0.49,  1.97) | 0.86(0.46,  1.60) | 1.04(0.71,  1.53) | 1.15(0.82,  1.61) |
| Preeclampsia | 0.84(0.34,  2.06) | 1.78(0.82,  3.88) | 1.67(0.15,  18.53) | 4.73(0.61,  36.87) | 1.72(0.75,  3.99) | 1.46(0.67,  3.18) |
| Preterm birth | 1.23(0.82,  1.86) | 1.40(0.95,  2.07) | 0.43(0.17,  1.10) | 0.94(0.48,  1.84) | 1.20(0.81,  1.77) | 1.40(0.99,  1.96) |
| PROM | 1.21(0.90,  1.64) | 1.61(1.21,  2.14) | 0.97(0.57,  1.66) | 1.07(0.67,  1.71) | 1.03(0.78,  1.37) | 1.14(0.89,  1.46) |
| TD | 0.76(0.57,  0.99) | 1.18(0.92,  1.51) | 0.86(0.51,  1.46) | 1.25(0.80,  1.94) | 1.07(0.80,  1.43) | 1.49(1.16,  1.91) |
| PROM | 1.21(0.90,  1.64) | 1.61(1.21,  2.14) | 0.97(0.57,  1.66) | 1.07(0.67,  1.71) | 1.03(0.78,  1.37) | 1.14(0.89,  1.46) |

Note: GDM: gestational diabetes mellitus; FGR: fetal growth restriction; PIH: pregnancy-induced hypertension; TD: thyroid dysfunction; LBW: low birth weight; PPH: postpartum hemorrhage; PROM: premature rupture of membranes.
